# Hydrogen Evolution Reaction Activity in Mo_2_TiC_2_T*x* MXene Derived from Mo_2_TiAlC_2_ MAX Phase: Insights from Compositional Transformations

**DOI:** 10.1021/acscatal.4c04099

**Published:** 2024-10-02

**Authors:** Jan Luxa, Petr Kupka, Fedor Lipilin, Jiří Šturala, Amutha Subramani, Petr Lazar, Zdeněk Sofer

**Affiliations:** † Department of Inorganic Chemistry, University of Chemistry and Technology, Technická 5, 166 28 Prague, Czech Republic; ‡ Regional Centre of Advanced Technologies and Materials, The Czech Advanced Technology and Research Institute (CATRIN), Palacký University Olomouc, Šlechtitelů 27, 779 00 Olomouc, Czech Republic

**Keywords:** MAX phases, MXenes, hydrogen evolution reaction, 2D materials, electrocatalysis

## Abstract

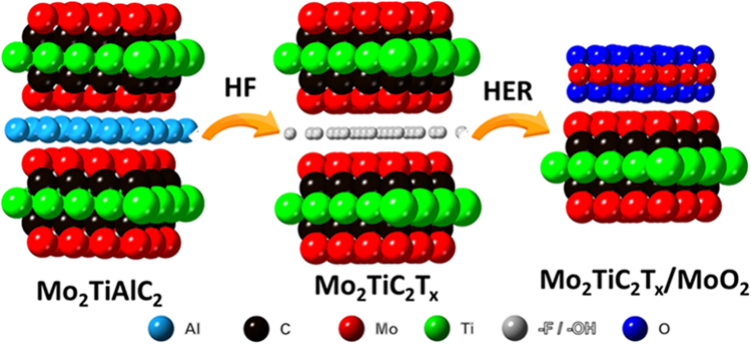

MAX phases represent a crucial building block for the synthesis of MXenes, which constitute an intriguing class of materials with significant application potential. This study investigates the catalytic properties of the Mo_2_TiAlC_2_ MAX phase and the corresponding Mo_2_TiC_2_T_*x*_ MXene for the hydrogen evolution reaction (HER). Characterization by X-ray diffraction (XRD), scanning electron microscopy (SEM), energy-dispersive spectroscopy (EDS), and X-ray photoelectron spectroscopy (XPS) revealed that despite the presence of secondary phases, the HER catalytic activity is primarily influenced by the MAX phase and its derived MXene. Interestingly, the catalytic activity of the MXene improves over time, attributed to the formation of MoO_2_ as identified by XPS. This work enhances the understanding of MXene-based materials for electrochemical applications, highlighting crucial structural and chemical transformations that optimize their performance in energy conversion technologies.

## Introduction

The consumption of energy in modern society plays a crucial role that needs to be addressed in a feasible and sustainable way. Currently, the majority of energy is produced via fossil fuels, which is a substantial burden from an ecological standpoint.^1^ The hydrogen economy has been proposed as an alternative for generating clean energy.^2^ However, a major challenge lies in the mass production of hydrogen from nonfossil fuel sources.^3^ The hydrogen evolution reaction (HER) is an electrochemical heterogeneous reaction that can achieve this goal if certain issues are addressed properly. A major drawback of HER is the need for an appropriate catalyst, as this reaction suffers from slow kinetics.^4^ The traditional state-of-the-art catalysts are based on Pt metal, which presents problems due to its scarcity and high cost. Over the last few decades, scientists have been pursuing alternatives based on abundant, nonprecious resources with the aim of achieving catalytic activity similar to that of Pt metal. Numerous efforts have been made on this topic, and various classes of materials including, but not limited to, transition and post-transition-metal chalcogenides, transition-metal phosphides, nitrides, carbides, carbon-based materials, pnictogens, and others have been tested.^5^ Among these, MXenes—functional derivatives of MAX phases—have attracted increasing attention for their potential in HER applications.^6^

MAX phases are layered compounds with the general formula M_*n*+1_AX*_n_*, where M represents an early transition metal (Ti, V, Zr, Mo, *etc*.), A is a non-transition metal (typically from groups 13 and 14, *e.g*., Al, Ga, *etc*.), and X is carbon and/or nitrogen.^7^ Based on their stoichiometry, these materials can be further divided into 211, 312, and 413 phases with the 211 (*e.g*., Ti_2_AlC) and 312 (*e.g*., Ti_3_AlC_2_) phases being the most extensively studied.^7^ What makes these materials intriguing is their physical and chemical properties. Thanks to their distinctive electronic structure, MAX phases combine the properties of metallic and ceramic materials. This includes high electrical and thermal conductivities, high stiffness, machinability as well as resistance to shock, oxidation, and, in some cases, creep.^8^

While MAX phases are fascinating materials, it is MXenes that have captured the attention of scientists and spurred significant research advancements in the field. MXenes are functional derivatives of MAX phases, characterized by the general formula M_*n*+1_X*_n_*T_*x*_, where T_*x*_ represents surface termination groups such as –F, –O, –H, or −OH.^9^ MXenes are typically prepared from MAX phases through selective etching of the A layer using concentrated HF or *in situ* generated HF from LiF and HCl. Density functional theory (DFT) calculations for Ti_3_AlC_2_ have shown that the exfoliation process begins with HF insertion into the edges of the MAX phase, followed by HF dissociation and subsequent termination of edge Ti atoms by −F or −H bonds.^9^ When an aqueous HF solution is used as the exfoliation agent, −OH terminations are also possible. Alternative exfoliation techniques, including alkali etching, electrochemical etching, and water-free etching, are also known, but fluoride etching remains the most widely used method.^10^

These exfoliation methods not only determine the structural properties of MXenes but also play a crucial role in influencing their catalytic performance, particularly in applications such as the HER. In terms of HER activity, MAX phases remain largely unexplored, mainly due to their low intrinsic activities.^11^ MXenes, on the other hand, have shown great promise in this field. Several approaches can be taken to enhance the catalytic activity of MXenes. One method is to control the surface termination, where it has been found that −O-terminated MXenes generally possess the highest catalytic activity.^12^ Another feasible method is to employ doping with low concentrations of highly active metals such as Pt.^13^ While such materials can achieve high HER activity, the use of noble metals does not address the issue of finding non-precious-metal alternatives. Even the synthesis of fine nanostructures, including nanodots or nanoribbons, can yield good results, achieving overpotentials as low as 169 mV at 10 mA cm^–2^.^14^ Despite the progress made in the field of MXenes for HER, a glaring issue remains: the vast majority of reports deal with Ti-based MXenes, while other materials could still offer significant application potential.

While MXenes derived from Ti-based MAX phases have been extensively studied for various applications, including hydrogen evolution reaction (HER) catalysis, Mo-based MAX phases, and their MXene derivatives remain largely underexplored. Notably, Mo_2_TiAlC_2_ is a recently synthesized MAX phase, and its conversion to Mo_2_TiC_2_T*_x_* MXene presents a unique opportunity to study a less common but potentially highly active material.

In this study, we synthesized Mo_2_TiAlC_2_ and its corresponding MXene Mo_2_TiC_2_T_*x*_ by using HF etching at room temperature. Both materials were analyzed by X-ray diffraction (XRD), scanning electron microscopy (SEM), energy-dispersive spectroscopy (EDS), transmission electron microscopy (TEM), thermogravimetric analysis (TGA), inductively coupled plasma optical emission spectroscopy (ICP-OES), and X-ray photoelectron spectroscopy, followed by an evaluation of their catalytic activity for the hydrogen evolution reaction. During extended HER testing, we observed that the catalytic performance of the MXene initially improved substantially before declining. XPS measurements taken at various points along the chronoamperometric curve revealed that MoO_2_ is formed on the surface of the material, which appears to be the primary factor contributing to the catalytic enhancement. This oxidation process is also accompanied by a reduction in the number of surface terminations introduced during exfoliation. To the best of our knowledge, this is the first study on Mo_2_TiAlC_2_-derived MXenes, offering new insights into the dynamic surface chemistry and its impact on catalytic performance.

## Results and Discussion

### Structural and Compositional Characterization

**Figure 1:**
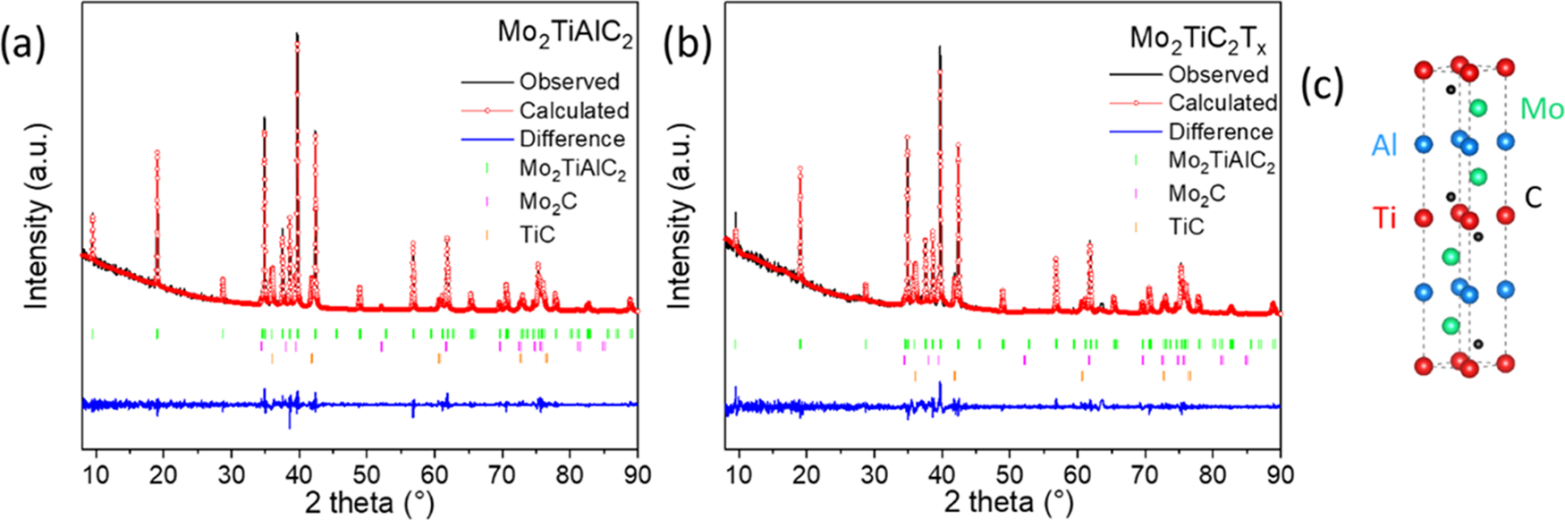
X-ray diffractograms of (a) Mo_2_TiAlC_2_ and (b) Mo_2_TiC_2_T_*x*_ together with (c) the structure of Mo_2_TiAlC_2_.

A MAX phase from the M_3_AX_2_ family, specifically Mo_2_TiAlC_2_, was synthesized and subsequently exfoliated into MXene Mo_2_TiC_2_T_*x*_, using HF etching to investigate the hydrogen evolution reaction activity of the exfoliated MXene. Notably, this MAX phase was recently synthesized for the first time, with the structure being stable only when Ti is sandwiched between two Mo layers.^15^ The structure of Mo_2_TiAlC_2_ is illustrated in Figure 1c. Interestingly, the HER activity of this MAX phase and its MXene has remained largely unexplored.

We first conducted an X-ray diffraction (XRD) analysis on the bulk phase to assess the presence of any impurities. As shown in Figure 1a, three phases were identified in the X-ray diffractogram: the Mo_2_TiAlC_2_ MAX phase along with the secondary phases Mo_2_C and TiC. The quantification of these secondary phases by XRD was achieved through Rietveld analysis, as seen in Figure 1a. The weight percentages obtained by Rietveld analysis (χ^2^ =1.16) were 74.34% for Mo_2_TiAlC_2_, 2.84% for Mo_2_C, and 22.82% for TiC, indicating that TiC is a major secondary phase. However, as will be demonstrated later in the paper, the HER activity of TiC is negligible compared to that of the exfoliated Mo_2_TiC_2_T*_x_*.

Following the analysis of the bulk MAX phase, we turned our attention to exfoliated MXene, with the results presented in Figure 1b. For the Rietveld analysis, the structural model of Mo_2_TiAlC_2_ was applied to the MXene. Similar to the parent phase, the etched material contained Mo_2_C and TiC as secondary phases. The composition of the material remained largely unchanged (χ^2^ = 1.28), with weight percentages of 73.25, 2.95, and 23.80% for Mo_2_TiAlC_2_, Mo_2_C, and TiC, respectively. At first glance, these results might suggest a low degree of etching. However, further analyses using energy-dispersive spectroscopy (EDS) and X-ray photoelectron spectroscopy (XPS) revealed a reduction in the Al content, alongside the presence of fluorine within the sample. This indicates that Al etching did occur, particularly on the surface of the material, as is discussed in further sections.

**Figure 2:**
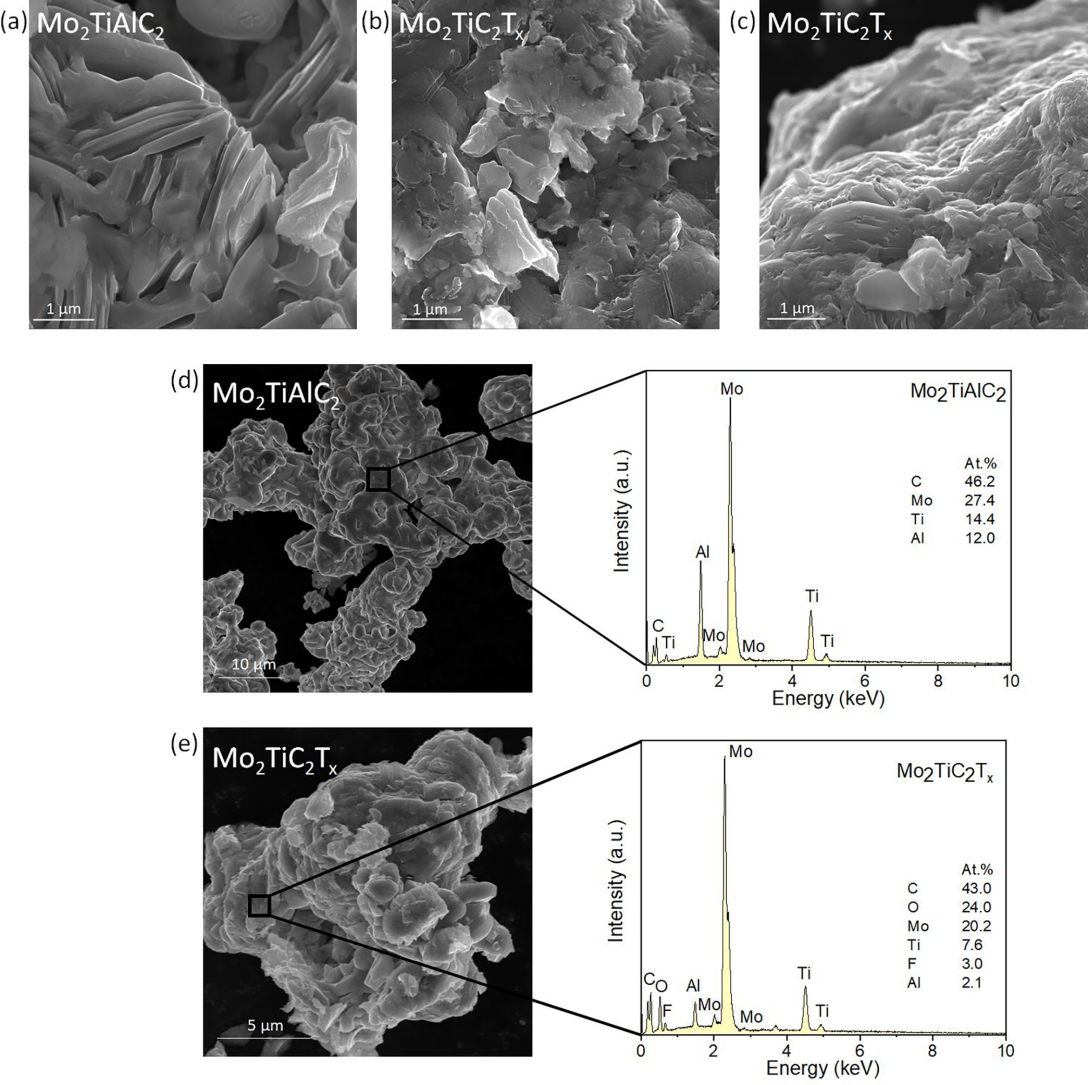
Scanning electron microscopy images of (a) Mo_2_TiAlC_2_ and (b, c) Mo_2_TiC_2_T_*x*_. SEM image with energy-dispersive spectra for (d) Mo_2_TiAlC_2_ and (e) Mo_2_TiC_2_T_*x*_.

Subsequent to the XRD and composition analyses, we examined the samples using scanning electron microscopy (SEM), with the results shown in Figure 2. The bulk Mo_2_TiAlC_2_ sample (Figure 2a) displayed the typical layered structure characteristic of MAX phases with crystallite sizes in the micrometer range. In contrast, the HF-etched Mo_2_TiC_2_T_*x*_ (Figure 2b,c), revealed a notable reduction in crystallite size, with sheets measuring below 1 μm. This sample also exhibited significant particle agglomeration, as most of the analyzed areas consisted of larger chunks formed by smaller, exfoliated pieces.

To complement our SEM observations, we performed energy-dispersive spectroscopy (EDS) on both the bulk and the exfoliated samples. Figure 2d shows the SEM image of the Mo_2_TiAlC_2_ sample, highlighting the area where the EDS spectrum was collected along with the corresponding spectrum. The measured metal ratio for Mo/Ti/Al was 1.91:1.00:0.83, which is in close agreement with the expected theoretical value of 2:1:1. Notably, the carbon content was found to be higher than expected, likely due to the inherent high uncertainty of EDS when quantifying light elements like carbon. Additionally, the absence of oxygen in the EDS analysis is significant, indicating that the sample remains unoxidized—a notable finding given the high reactivity of elements such as Ti and Al. These results provide further evidence of the pristine nature of the MAX phase prior to etching, setting a clear baseline for understanding the changes induced by HF treatment.

For the Mo_2_TiC_2_T*_x_* sample, we observed a significant reduction in Al concentration down to 2.1 atom %, confirming the effective removal of Al during the etching process. To further verify the metal content, we employed inductively coupled plasma optical emission spectrometry (ICP-OES), which revealed a metal ratio of Mo/Ti/Al as 1.85:1.00:0.67 and an Al concentration of approximately 11.8 atom %. The discrepancies observed among EDS, ICP-OES, and X-ray photoelectron spectroscopy (XPS) results stem from the varying depth sensitivities of these techniques: ICP-OES reflects the overall bulk composition, EDS analyzes elements from several micrometers in depth, and XPS provides surface-sensitive measurements. XPS analysis further confirmed that Al was absent on the surface of the Mo_2_TiC_2_T*_x_* sample, suggesting that any remaining Al was confined to the unexfoliated portions of the material. Additionally, both oxygen and fluorine were detected, indicating successful modification of the MXene surface with the –F and –OH termination groups. These findings highlight the changes in surface chemistry induced by etching, which are crucial for understanding the enhanced catalytic performance discussed in subsequent sections.

**Figure 3:**
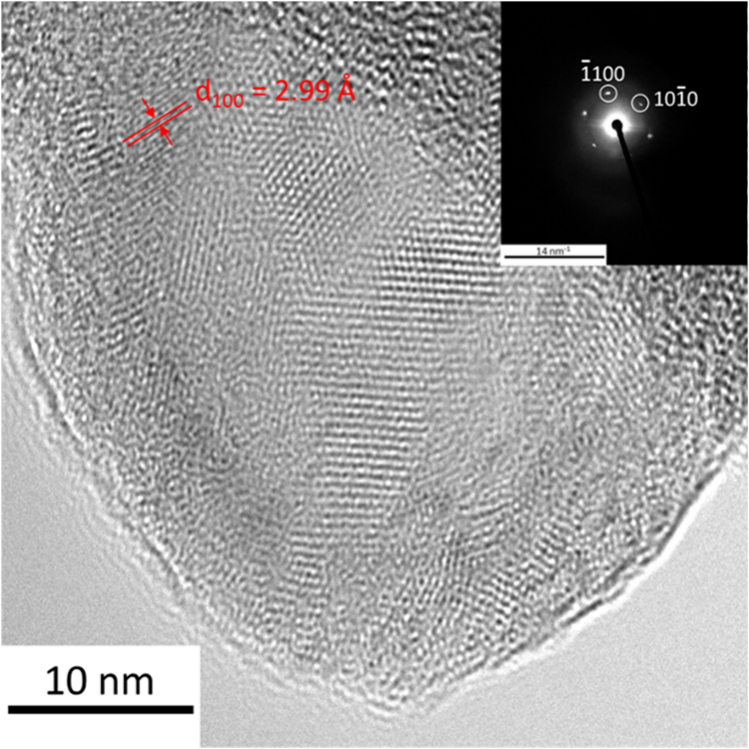
High-resolution transmission electron microscopy image of the Mo_2_TiC_2_T_*x*_ sample together with a selected area electron diffraction pattern. The SAED pattern was collected along the [00l] direction.

The structure of Mo_2_TiC_2_T*_x_* was further examined by using high-resolution transmission electron microscopy (HR-TEM) combined with selected area electron diffraction (SAED), as shown in Figure 3. The HR-TEM image of a single Mo_2_TiC_2_T*_x_* sheet along the [00l] direction reveals characteristic plane fringes, specifically (100) planes, with their interlayer distances highlighted in the image. Notably, areas of disorder within the sheet, particularly along the edges, suggest that the pristine structure of the original MAX phase has been partially disrupted due to the introduction of functional groups during the HF etching process. Despite these disruptions, the overall symmetry of the phase remains consistent with the parent MAX phase, as evidenced by the hexagonal SAED pattern displayed in the top right corner of Figure 3. This finding aligns well with the XRD results, further confirming that the core structural framework of the material is preserved even after exfoliation and surface modification.

The presence of surface functional groups and the thermal stability of Mo_2_TiC_2_T*_x_* were further investigated by using thermogravimetric analysis (TGA), with results presented in Figure S1. While TGA characterization of Mo_2_TiC_2_T*_x_* is currently absent in the literature, similar analyses have been reported for Mo_2_CT_x_ synthesized from Mo_2_Ga_2_C by the Gogotsi group.^16^ The TGA curve of Mo_2_TiC_2_T_*x*_ demonstrates thermal stability up to approximately 100 °C, after which a weight loss (1 wt %) occurs until around 200 °C. Based on previous reports for Mo_2_CT_*x*_, this initial weight loss is likely due to the release of H_2_O molecules trapped between the MXene layers.^16^ Beyond this point, the weight loss proceeds more gradually up to about 500 °C, which can be attributed to the release of additional water and the decomposition of −OH groups. This observation aligns with the tendency of Mo-based MXenes to have predominantly O-type terminations.^16,17^ The final weight loss around 500 °C is likely caused by the release of CO, resulting from the reaction between carbon- and oxygen-terminated groups present on Mo_2_TiC_2_T*_x_*. Additionally, −F terminations may decompose within the 200–700 °C range, contributing to the overall weight loss observed during this temperature interval.^16^ The slight variations in temperature profiles compared to those in previous reports are likely due to differences in the specific compositions of the materials studied.

**Figure 4:**
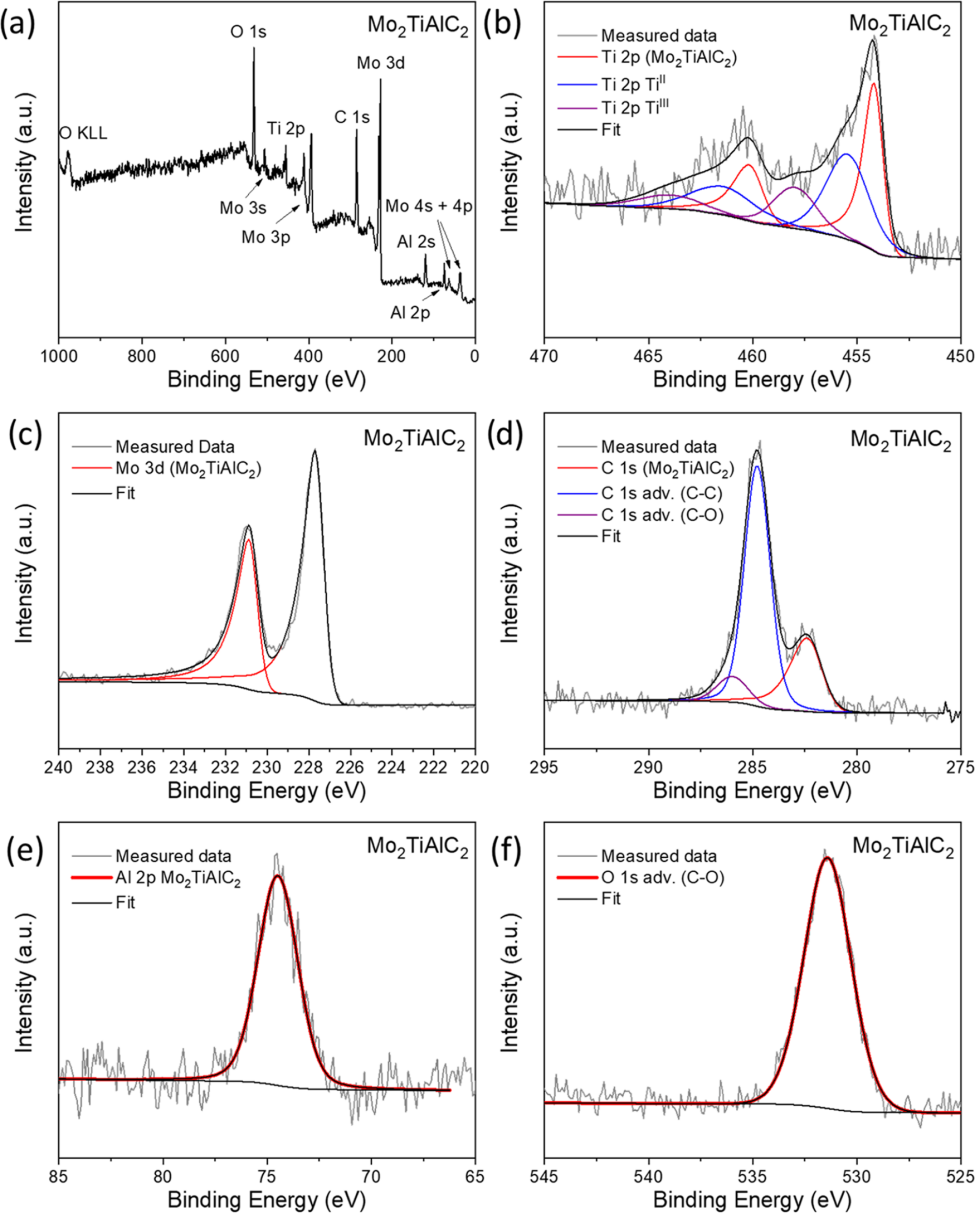
X-ray photoelectron spectra of Mo_2_TiAlC_2_ sample. (a) Survey spectrum; (b) Ti 2p spectrum; (c) Mo 3d spectrum; (d) C 1s spectrum; (e) Al 2p spectrum; and (f) O 1s spectrum.

Finally, X-ray photoelectron spectroscopy (XPS) measurements were conducted to complete the structural and compositional analysis of the MAX phase and the MXene. Figure 4 presents the spectra associated with the Mo_2_TiAlC_2_ sample with detailed peak fitting parameters summarized in Table S1. The survey spectrum shown in Figure 4a confirms the surface purity of the sample as only the expected elements were detected. Figure 4b illustrates the deconvolution of the Ti 2p core-level spectrum, which was performed based on a previous XPS study of Mo_2_TiAlC_2_.^18^ The first doublet with an asymmetric peak shape was attributed to Mo_2_TiAlC_2_. Two more components attributed to Mo_2_Ti^II^AlC_2_ and Mo_2_Ti^III^AlC_2_ were also identified in the spectrum, consistent with prior reports.^18^ For Mo 3d, displayed in Figure 4c, a single doublet with an asymmetric peak line shape was observed, corresponding exclusively to Mo_2_TiAlC_2_. The presence of TiC and Mo_2_C phases in Ti 2p and Mo 3d phases could not be exclusively determined due to the overlapping peak positions of these phases with Mo_2_TiAlC_2_, specifically at approximately 455 eV for TiC and 227.8 eV for Mo_2_C.^18,19^ The C 1s spectrum in Figure 4d clearly shows the presence of carbidic carbon, marked by a peak at around 282.4 eV, along with peaks attributed to adventitious carbon. The Al 2p spectrum shown in Figure 4e, was deconvoluted into a single peak, which was attributed to the Mo_2_TiAlC_2_ phase. Finally, the O 1s peak in Figure 4f mostly originates from adventitious contamination as most metal oxides exhibit peaks located at or below 530 eV.^20^

**Figure 5:**
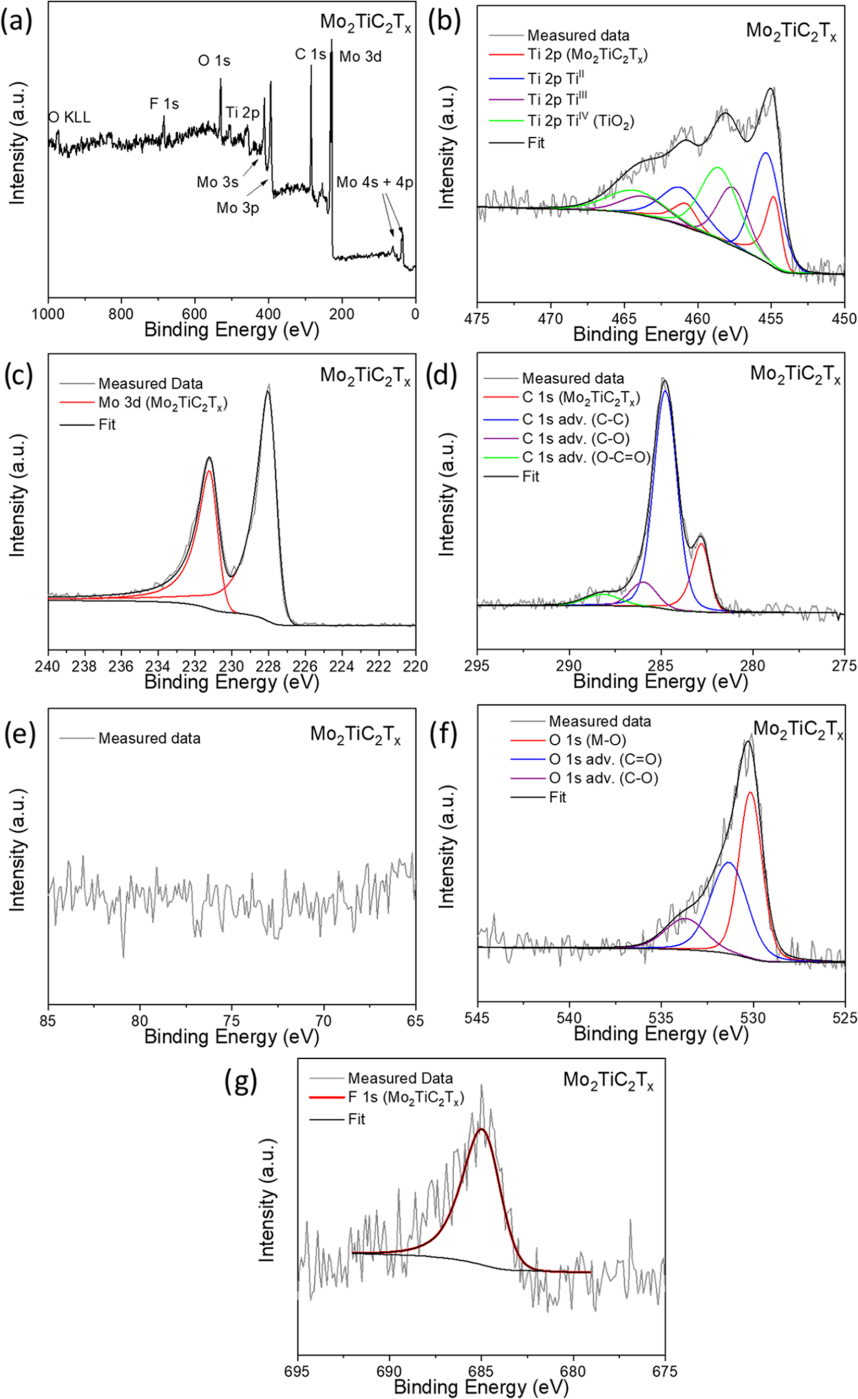
X-ray photoelectron spectra of Mo_2_TiC_2_T_*x*_ sample. (a) Survey spectrum; (b) Ti 2p spectrum; (c) Mo 3d spectrum; (d) C 1s spectrum; (e) Al 2p spectrum; (f) O 1s spectrum; and (g) F 1s spectrum.

The XPS analysis for the Mo_2_TiC_2_T*_x_* sample is presented in Figure 5, with detailed peak fitting parameters provided in Table S2. Several notable differences between the MAX phase and exfoliated MXene are evident. First, the Mo_2_TiC_2_T_*x*_ sample shows a complete absence of Al, and concurrently, an F 1s peak appears (Figure 5a,e,g). These observations confirm the effective removal of Al from the surface and the introduction of −F functional groups. In contrast, the Mo 3d (Figure 5c) and C 1s (Figure 5d) spectra closely resemble those of the original MAX phase. However, a closer examination of the Mo 3d spectrum, along with data in Table S2, reveals a slight shift in the binding energy of Mo peaks to higher values (0.3 eV). This shift can be attributed to the presence of electronegative −O and −F terminations.^17^ Significant changes are also observed in the Ti 2p spectrum, which shows new peak doublets at 458.6 and 464.5 eV for Ti 2p_3/2_ and Ti 2p_1/2_, respectively. This doublet corresponds to TiO_2_, indicating that Ti undergoes oxidation during the exfoliation process.^21^ This is also well demonstrated in the O 1s spectrum in Figure 5f where a shoulder at lower binding energies associated with the TiO_2_ can be observed.^20^ Finally, the F 1s spectrum displays a broad peak with a tail extending toward higher binding energies, suggesting the presence of multiple states (fitting with a single peak results in a full width at half-maximum (fwhm) of 2.36), which may be due to −F terminations associated with both Mo and Ti. Although detailed data for Mo_2_TiC_2_T*_x_* are limited, as F 1s spectra are rarely reported in the literature, this broadening is noteworthy. Notably, as will be shown later, the F 1s spectrum narrows and shifts slightly to lower binding energies in samples subjected to extended HER testing, likely indicating the removal of −F terminations from Mo atoms. Further discussion of this behavior can be found in later sections.

### Hydrogen Evolution Reaction

**Figure 6:**
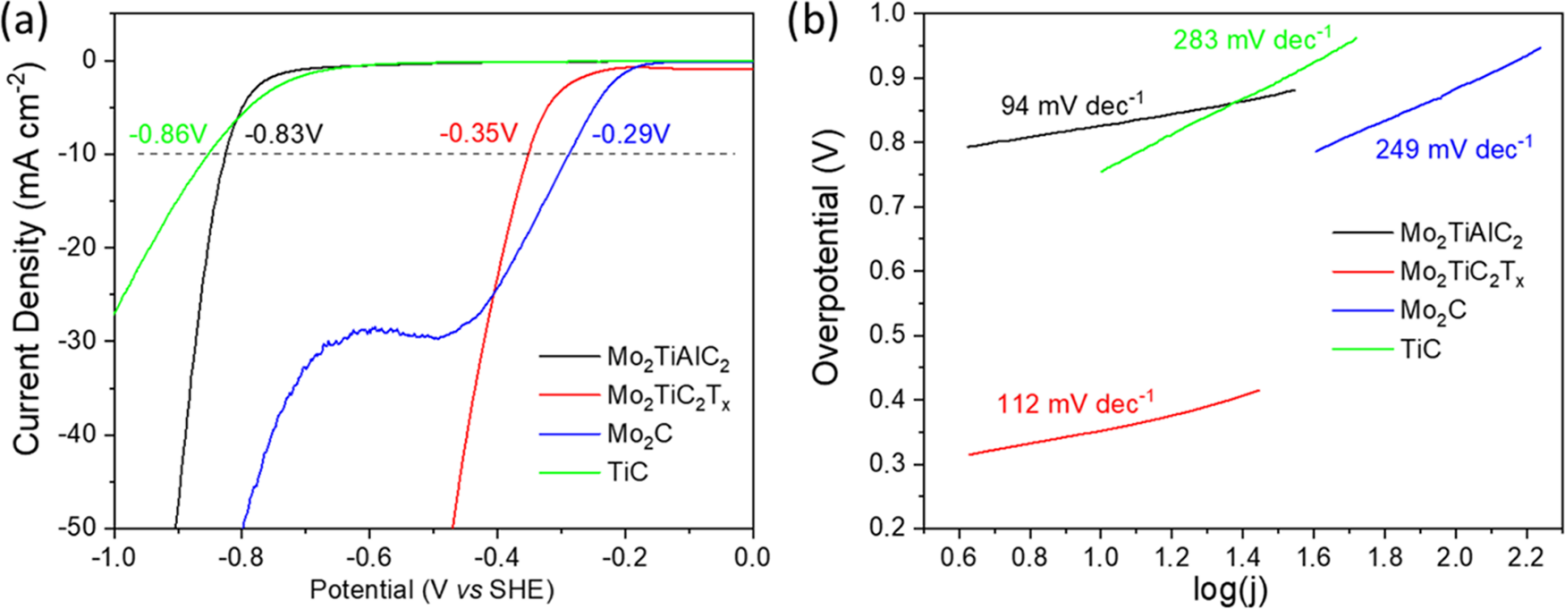
(a) Linear sweep voltammetry curves of Mo_2_TiAlC_2_, Mo_2_TiC_2_T_*x*_, TiC and Mo_2_C and (b) the corresponding Tafel slope curves.

With the thorough characterization of the materials complete, we proceeded to evaluate their electrocatalytic activity for the HER. The results of linear sweep voltammetry (LSV) are presented in Figure 6a, showing the performance of Mo_2_TiAlC_2_, Mo_2_TiC_2_T*_x_* along with TiC and Mo_2_C which were identified as secondary phases by XRD. The benchmark used for comparison was the standard overpotential at −10 mA cm^–2^ which is highlighted in the figure. It is immediately evident that the bulk Mo_2_TiAlC_2_ phase exhibits a relatively high overpotential of 0.83 V, while exfoliating into Mo_2_TiC_2_T*_x_* significantly reduces the overpotential to 0.35 V. This substantial decrease indicates that the introduction of −F and −OH functional groups during exfoliation markedly enhances the HER catalytic activity, consistent with findings reported for other MXenes. Further comparison with the LSV curve for TiC, the major secondary phase, underscores that the catalytic activity for both the bulk and exfoliated materials predominantly originates from the MAX phase and derived MXene, as TiC has a large overpotential of 0.86 V. The low content of Mo_2_C further suggests it is unlikely to significantly contribute to the overall HER activity. Additionally, the LSV curve for Mo_2_C appears somewhat misleading; it initially shows a reduction with a peak around −0.45 V vs SHE before an increase in the current density associated with HER, further confirming its minimal impact on the catalytic performance of the system.

Figure 6b displays the Tafel slope curves and Tafel slope values derived from the LSV data. The Tafel slope for Mo_2_TiAlC_2_ was determined to be 94 mV dec^–1^, while Mo_2_TiC_2_T*_x_* exhibited a slightly higher Tafel slope of 112 mV dec^–1^. Despite this increase, the much lower overpotential of the exfoliated Mo_2_TiC_2_T*_x_* clearly indicates superior HER activity compared with that of the bulk MAX phase. In contrast, the TiC and Mo_2_C phases displayed significantly larger Tafel slope values of 283 and 249 mV dec^–1^, respectively, further confirming their minimal contribution to the overall HER performance.

The stability test of Mo_2_TiC_2_T*_x_* was performed via chronoamperometry, as shown in Figure S2a. Long-term measurements revealed an intriguing behavior: the catalytic activity initially increased during the first 13 h, followed by a gradual decline, eventually returning to the original current density after approximately 53 h. These key points are highlighted in Figure S2a. To further investigate this phenomenon, electrochemical impedance spectroscopy (EIS) measurements were conducted at the beginning of the stability test, as well as after 13 and 53 h. The resulting impedance spectra, presented as Nyquist plots in Figure S2b, display a characteristic semicircle accompanied by an inductive loop in the low-frequency region. The semicircle corresponds to the double-layer capacitance and charge transfer resistance (*R*_CT_) associated with HER, while the inductive loop has been linked to the dynamic adsorption and desorption of hydrogen at the electrode surface.^22^

The equivalent circuit used to describe the system, shown in the inset of Figure S2b, was based on previous reports where similar inductive loops related to hydrogen adsorption were observed.^22^ The model consists of constant phase elements (CPE) representing the double-layer capacitance of inhomogeneous surfaces, an inductor (L) associated with hydrogen adsorption, and a series of resistances: *R*_S_ (solution resistance), *R*_CT_ (charge transfer resistance across the double layer), and *R*_L_ (resistance related to hydrogen adsorption). The impedance of the CPE is defined by the equation:
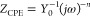
where *Y*_0_ is a time constant parameter, ω is the angular frequency, and *n* is a CPE exponent. The parameters obtained from fitting the equivalent circuit are summarized in Table S3. Among these, the most impactful parameter in assessing the overall catalytic activity is *R*_CT_, as it directly reflects the material’s ability to transfer charge during HER. Initially, the *R*_CT_ value was 32.75 Ω, which decreased to 22.47 Ω after 13 h, consistent with the increased current density observed in Figure S2a. However, after 53 h, the *R*_CT_ value rose to 51.22 Ω, correlating with the observed decline in catalytic activity.

Since the catalytic activity of materials is driven by the surface active site, XPS analysis was performed on Mo_2_TiC_2_T*_x_* after 13 and 53 h to identify the chemical changes responsible for the observed variations in catalytic performance. The photoelectron spectra for Mo_2_TiC_2_T*_x_* at 13 h are shown in Figure S3, with peak fitting details summarized in Table S4. Significant changes in surface chemistry were detected for Ti, Mo, and O, along with a noticeable decrease in fluorine concentration, which became evident only after high-resolution spectra were collected. Notably, the Ti 2p spectrum (Figure S3b) showed the complete disappearance of the TiO_2_ signal, suggesting the dissolution of TiO_2_ in the acidic H_2_SO_4_ solution used during measurements. A possible explanation is the formation of titanyl sulfate (TiOSO_4_), a known product from the production of TiO_2_ pigment from ilmenite ore (FeTiO_3_).^23^

More pronounced changes were observed in the Mo 3d spectrum (Figure S3c). The peak doublet initially attributed to the MAX phase diminished significantly, and several new peaks with complex spectral structures emerged. Comparison with the literature data indicated that the formation of MoO_3_ and MoO_2_ was the most plausible outcome. For MoO_3_, two components were identified with peak shapes and positions closely matching reported values.^24,25^ Despite the presence of doublets from Mo_2_TiC_2_T*_x_* and MoO_3_, it became clear that at least four components were required to achieve a reliable spectral fit. Previous studies have noted that MoO_2_ can exhibit complex spectral features, similar to those observed here, making precise determination of Mo oxides with mixed oxidation states challenging.^24,25^ In this analysis, one doublet was assigned to screened MoO_2_ electrons and another to unscreened MoO_2_ electrons. Unlike previous reports, no peaks corresponding to Mo^V^ oxides were observed, likely because such species typically form only after prolonged X-ray exposure of MoO_3_, which was not part of our experimental conditions.^24^ Furthermore, the potential formation of Mo-based Magnéli phases, which are nonstoichiometric Mo oxides, cannot be ruled out. However, recent HER studies on MoO_2_ have demonstrated increased catalytic activity with extended cycling, suggesting that the formation of MoO_2_ on the MXene surface likely contributes to the enhanced catalytic performance observed.^26^ Additionally, changes were also noted in the O 1s spectrum, with an apparent peak around 530 eV attributed to metal oxides.^24^ The observed decrease in F concentration, along with the narrowing of the F 1s spectrum (fwhm reduced from 2.36 to 1.47) and a shift to lower binding energies (from 685.1 to 648.8 eV), suggests that −F groups are gradually removed, most likely from the Mo surface. This replacement with oxide materials contributes to the overall chemical evolution of MXene during HER testing, as further supported by DFT calculations discussed in later sections.

The final set of photoelectron spectra for Mo_2_TiC_2_T*_x_* at 53 h is shown in Figure S4, with peak fitting details summarized in Table S5. The most notable change from the sample analyzed at 13 h is the near-total absence of F in the sample. The F 1s peak was missing from the survey spectrum, and only a very low-intensity signal was detected after multiple acquisitions of the F 1s region (Figure S4g). Due to the low signal, peak fitting for the F 1s spectrum was not performed, resulting in low confidence in any quantitative interpretation from this region. Additionally, there was a further increase in the concentration of MoO_2_ with a simultaneous decrease in the carbidic Mo component. These observations suggest that extended hydrogen evolution in acidic media promotes the gradual removal of −F functionalities as the MXene surface gets oxidized, predominantly forming MoO_2_. However, the observed decrease in catalytic activity also indicates a possible synergistic catalytic effect between the original MXene and the oxidized material, highlighting that the interplay between these phases may be crucial for optimizing the HER performance.

To further investigate the observed changes in catalytic activity and gain more detailed mechanistic insights, we performed density functional theory (DFT) calculations of the Gibbs free energy of adsorbed hydrogen (Δ*G*_H_) on the MXene surfaces. Based on our experimental findings, which detected O and F within the Mo_2_TiC_2_T*_x_* sample, we modeled two types of MXene surfaces: (1) MXene terminated with −OH groups and (2) MXene terminated with −F atoms. The calculations reveal that −OH-terminated MXene is highly reactive toward hydrogen. Depending on the initial adsorption site of the H atom on the surface, hydrogen either reacts to form a H_2_O molecule (Δ*G*_H_ = −2.47 eV) or dissociates one of the −OH groups to form H_2_ (Δ*G*_H_ = −2.26 eV). Both reactions lead to the removal of hydrogen from the surface, resulting in −O-terminated regions on the MXene surface. This suggests that −OH-terminated areas of the sample likely convert to −O-terminated regions during the initial phase of the HER. To assess the impact of this transformation on HER activity, we calculated the Δ*G*_H_ of −O-terminated MXene. Hydrogen preferentially adsorbs onto the surface oxygen atom. The resulting Δ*G*_H_ is −0.59 eV, indicating that −O-terminated regions are moderately active for HER. These findings suggest that the dynamic surface chemistry involving the transformation of −OH to −O termination plays a significant role in modulating the catalytic properties of MXene during HER.

For the −F terminated MXene, two competing processes were identified. In the first process, hydrogen binds to the hollow site between F atoms, directly interacting with the Mo atom in the surface layer. This process is nearly thermoneutral with a Δ*G*_H_ of −0.14 eV. In the second process, as the H atom approaches an F atom, HF is formed, leading to the detachment of the F atom from the MXene surface. This reaction has a Δ*G*_H_ of −0.09 eV, similar to that of hydrogen adsorption, suggesting that both processes occur simultaneously. These results indicate that −F-terminated MXene is HER active but also undergoes defluorination during the reaction, aligning with the experimentally observed decrease in fluorine content.

To further explore the impact of defluorination, we examined the behavior of the resulting bare Mo-terminated MXene surface. This surface binds hydrogen strongly, with Δ*G*_H_ values of −0.68, −0.56, −0.51, and −0.51 eV corresponding to 25, 50, 75, and 100% surface coverage by hydrogen, respectively. After full coverage by hydrogen (100%), further hydrogen adsorption occurs directly on top of the Mo atom and Δ*G*_H_ approaches thermoneutrality at 0.35 eV. Additionally, the bare Mo surface is prone to oxidation, forming MoO_2_ with the simultaneous release of H_2_, consistent with the MoO_2_ formation observed in XPS data.

Considering these findings, along with the presence of both −F- and −OH-terminated MXene in our samples, a complex interplay of reactions is evident, including hydrogen evolution, defluorination, −OH dissociation, and MoO_2_ formation. These processes occur simultaneously and influence each other, collectively contributing to the observed performance pattern of the MXene catalyst.

## Conclusions

In conclusion, this study provides valuable insights into the hydrogen evolution reaction (HER) catalytic activity of the Mo_2_TiAlC_2_ MAX phase and its derived Mo_2_TiC_2_T*_x_* MXene. X-ray diffraction (XRD) identified secondary phases Mo_2_C and TiC, with TiC being the more abundant. HF exfoliation resulted in partial delamination of the MAX phase and complete removal of surface-bound Al, which was replaced by −F, −OH, or −O functional groups, as confirmed by energy-dispersive spectroscopy (EDS) and X-ray photoelectron spectroscopy (XPS). The HER catalytic activity was primarily attributed to the Mo_2_TiAlC_2_ phase, as the secondary phases displayed a significantly lower performance with higher overpotentials.

Exfoliation into Mo_2_TiC_2_T*_x_* MXene significantly enhanced HER activity, reducing the overpotential from 0.83 to 0.35 V. Chronoamperometry testing revealed an initial increase in activity followed by a gradual decline, correlating with the formation of MoO_2_ observed in XPS analysis. This transformation was accompanied by a decrease in fluorine content, indicating a shift toward MoO_2_ formation. Further XPS analysis post decline showed continued fluorine removal and increased MoO_2_ content, suggesting a potential synergistic catalytic effect between MoO_2_ and the functionalized MXene in catalysis. DFT calculations provided deeper mechanistic insights, showing that multiple competing processes—including hydrogen evolution, defluorination, −OH dissociation, and MoO_2_ formation—occur simultaneously. These findings underscore the importance of comprehensive characterization during continuous catalytic testing to fully understand material transformations and optimize the performance in electrochemical applications. This work highlights the complex interplay of surface chemistry and structural evolution in MXene-based catalysts, providing a pathway for enhancing the HER performance in future studies.

## Materials and Methods

### Materials

Mo (99.95% purity, −300 mesh) was purchased from Beijing Metallurgy and Materials Technology Co., TiH_2_ (99.7%, −325 mesh) was purchased from Beijing Metallurgy and Materials Technology Co., Al (99.7%, −325 mesh) was purchased from STREM, USA, and graphite (99%, −325 mesh) was purchased from Sigma-Aldrich, Czech Republic. Sulfuric acid and ethanol were obtained from Penta Chemicals, Czech Republic.

### Synthesis

Bulk Mo_2_TiAlC_2_ was synthesized by mixing Mo (65.39g), TiH_2_ (17.00g), Al (10.11g), and graphite (8.19g) giving a ratio of Mo/Ti/Al/C equal to 2:1:1.1:1. After homogenization by mixing, the reactants were placed in an Al_2_O_3_ crucible after which the furnace was evacuated and purged by Ar several times. The mixture was then heated to 1500 °C at a rate of 3 °C min^–1^ followed by a 2 h dwell at this temperature. Finally, the mixture was cooled to room temperature at a rate of 3 °C min^–1^.

The Mo_2_TiC_2_T_*x*_ MXene was synthesized by HF etching. For this purpose, 25 mL of 40 wt % aqueous HF solution was placed in a Teflon vessel after which 1 g of Mo_2_TiAlC_2_ was added slowly. The mixture was kept at room temperature for 6 days under continuous stirring. After that, excess HF was removed by centrifugation with deionized water. This process was repeated until a pH of ∼7 was reached. Finally, the MXene was dried in a vacuum oven at 40 °C for 24 h.

### Characterization Techniques

X-ray diffraction was carried out on a Bruker D8 Dis-cover (Bruker) with Cu X-ray source (λ= 0,15418 nm, *U* = 40 kV, *I* = 40 mA). Diffractograms were collected in a range from 5 to 90° with a step of 0.02° and step time of 0.5s. Phase identification was carried out in HighScore Plus software. For Rietveld analysis, the FullProf software package.

The morphology of materials was investigated by using scanning electron microscopy (SEM) with a field emission gun (FEG) electron source (Tescan Maya microscope). The samples were placed on carbon tape and measured using a 15 kV acceleration beam voltage. The composition of the samples was determined by means of energy-dispersive spectroscopy (EDS) analyzer (X-MaxN) with a 150 mm^2^ SDD detector (Oxford instruments). Data were evaluated by using AZtecEnergy software.

Transmission electron microscopy (TEM) micrographs were obtained using a JEOL JEM-1010 instrument operating at an accelerating voltage of 80 kV. Pictures were taken by SIS MegaView III digital camera (Soft Imaging Systems) and analyzed by AnalySIS version 2.0 software. To prepare the sample for TEM, the Mo_2_TiC_2_T_*x*_ was sonicated in water in order to obtain a suspension of the material.

Thermogravimetric analysis was carried out using a Themys TGA (SETARAM, Caluire, France) at a temperature range between 30 and 800 °C and a heating rate of 10 K min^–1^. The instrument was purged with argon for 1 h before the measurement started and to equilibrate the temperature at 30 °C. Argon was used as a carrier gas with a flow rate of 100 mL min^–1^ (heating rate 10 K min^–1^). About 5 mg of Mo_2_TiAlC_2_ or Mo_2_TiC_2_T_*x*_ was used for the analysis.

ICP-OES measurements were performed with a Spectro ARCOS (SPECTRO Analytical Instruments). The spectrometer used the Paschen–Runge configuration with an optimized Rowland circle polychromator, measuring simultaneously in the broad spectral range of 130–770 nm using 32 linear CCD detectors. The detection limits are at parts per billion (by mass) levels for the elements determined. For the measurements, 4 mg of the Mo_2_TiC_2_T_*x*_ was decomposed under microwave radiation in a mixture of H_2_O_2_, HNO_3_, HF a HClO_4_ and then determined.

X-ray photoelectron spectroscopy was carried out on a Phoibos 100 (Specs, Germany) with a monochromatic Al source (*K*_α1_ = 1486.7 eV). For bulk and exfoliated MXene, the samples were placed onto Cu conductive tape. Samples analyzed after HER activity measurements were measured on screen-printed electrodes. For survey spectra, an *E*_pass_ = 50 eV with a step of 1 eV was used, while for high-resolution core-level spectra, an *E*_pass_ = 20 eV with a step of 0.1 eV was used. Compensation using a flood gun was not necessary due to the good conductivity of the samples.

For electrochemical measurements, Autolab PGSTAT 204 (Metrohm, Czech Republic) was used. For the hydrogen evolution reaction, we used glassy carbon (GC) electrode, graphite rod, and saturated calomel electrode as working, counter, and reference electrodes, respectively. The GC electrode was modified with 7.5 μL aliquot of suspension (1:1 volume ratio of H_2_O and EtOH, concertation of 5 mg mL^–1^) resulting in a catalyst loading of 0.53 mg cm^–2^, followed by drying in air. The reaction was performed in a 0.5 M H_2_SO_4_ solution purged with argon. Linear sweep voltammograms were carried out at a scan rate of 10 mV s^–1^. The potential was converted with regard to the reversible hydrogen electrode according to the equation
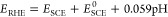
where *E*_SCE_ is the measured potential and *E*_SCE_^0^ is a standard reduction potential of the calomel electrode. All measurements are reported without *iR* compensation.

For stability measurements, 0.1 μL of Nafion (3 wt % solution) to 1 mL of the aforementioned suspension was added to ensure adhesion of the material to the GC electrode. For stability XPS measurements, the MXene was drop-cast onto screen-printed electrodes without the addition of Nafion to prevent surface contamination. L-shaped electrodes were used to prevent the delamination of the material from the electrode. Before XPS measurements, the screen-printed electrodes were washed with deionized water several times to remove residual H_2_SO_4_.

Electrochemical impedance spectroscopy measurements were performed in an experimental setup identical to that described in previous paragraphs. A constant DC voltage of ca. −0.35 V vs RHE was applied with a 10 mV sinusoidal AC voltage amplitude. A frequency range of 0.1 Hz to 100 kHz was used. Before the first measurement of the Mo_2_TiC_2_T_*x*_, a 5 min stabilization period with DC of −0.35 V vs RHE was used. Before collecting the EIS spectra after 13 and 53 h, the voltage was kept at a constant value of −0.35 V vs RHE for the respective amount of time. The data were evaluated in EIS analyzer software.

### DFT Calculations

DFT calculations were performed using the projector-augmented wave method implemented in the Vienna Ab initio Simulation Package (VASP).^27^ The energy cutoff for the plane-wave expansion was set to 500 eV. We used optimized van der Waals functional optB86b-vdW functional, which provides a balanced description of van der Waals as well as covalent and ionic bonding.^28^ We optimized the crystal structure of Mo_2_TiC_2_T*_x_* MXene for two surface terminations, T = −F and T = −OH. We obtained the lattice parameters *a* = 2.95 and *a* = 2.98 Å, respectively. The surface (basal plane) was modeled by a 2 × 2 supercell slab in connection with 6 × 6 × 1 *k*-point sampling. The individual slabs were separated by 15 Å of vacuum. The Gibbs free energy of the adsorption of atomic hydrogen (Δ*G*_H_) was used to describe the HER activity of the catalyst.^29^ The Δ*G*_H_ of an ideal catalyst for the HER should be as close to zero as possible. The Gibbs free energy of the adsorption atomic hydrogen (Δ*G*) was calculated as
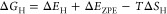
where Δ*S*_H_ accounts for the difference in zero-point energy, and entropy between the adsorbed hydrogen and hydrogen in the gas phase. These thermal corrections were found to be independent of particular adsorption site, and thus can be described as a thermal correction constant of 0.29 eV.^29^ The differential energy of adsorption Δ*E*_H_ was calculated from calculated DFT energies as
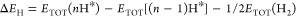
where *E*_TOT_(*n*H*) stands for the total energy of *n* hydrogen atoms adsorbed on the surface of MXene and *E*_TOT_(H_2_) for the total energy of the H_2_ molecule used as the reference state.

## Data Availability

The data sets generated during and/or analyzed during the study are accessible via the Zenodo repository: https://zenodo.org/records/12699958.

## Supporting Information Available

Tables with XPS fitting details (Tables S1, S2, S4, S5); Thermogravimetric analysis (Figure S1); Chronoamperometric curve (Figure S2) and electrochemical impedance spectroscopy curves (Figure S2); and X-ray photoelectron spectra of Mo_2_TiC_2_T_*x*_ 13 h sample (Figure S3) and Mo_2_TiC_2_T_*x*_ 53 h sample (Figure S4) (PDF)
