# Supplementary material for: Hydrogen Evolution Reaction Activity in Mo2TiC2Tx MXene Derived from Mo2TiAlC2 MAX Phase: Insights from Compositional Transformations
Source: ACS Catal. 2024 Oct 2;14(20):15336–47. doi: 10.1021/acscatal.4c04099 (PMC11494504; doi:10.1021/acscatal.4c04099)
Supplement: Supplementary file 1 — cs4c04099_si_001.pdf [file cs4c04099_si_001.pdf]

Supporting information

Hydrogen Evolution Reaction Activity in $Mo_2TiC_2T_x$ MXene Derived from Mo_2TiAlC_2 MAX Phase: Insights from Compositional Transformations

Jan Luxa [a,], Petr Kupka [a], Fedor Lipilin [a], Jiří Šturala [a], Amutha Subramani [a], Petr Lazar [b] and Zdeněk Sofer [a,*]*

[a] Department of Inorganic Chemistry, University of Chemistry and Technology, Technická 5, 166 28, Prague, Czech Republic

[b] Regional Centre of Advanced Technologies and Materials, The Czech Advanced Technology and Research Institute (CATRIN), Palacký University Olomouc, Šlechtitelů 27, 779 00 Olomouc, Czech Republic

Corresponding Author(s)

Jan Luxa: jan.luxa@vscht.cz

Zdeněk Sofer: zdenek.sofer@vscht.cz

KEYWORDS: MAX phases, MXenes, hydrogen evolution reaction, 2D materials, electrocatalysis

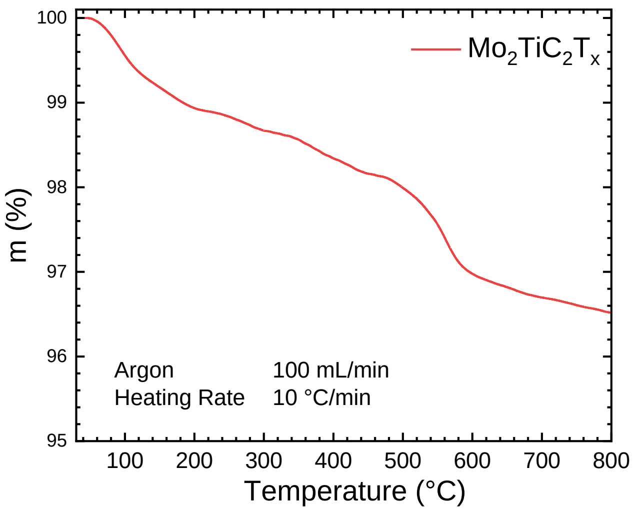

Figure S1. Thermogravimetric analysis curve of the $Mo_2TiC_2T_x$ sample performed in an Ar atmosphere at a 100 ml/min flow and a heating rate of 10 °C/min.

Table S1. Peak positions, full-width at half maximum (FWHM), concentration and assignement for XPS data for sample Mo_2TiAlC_2.

Region	BE (eV)	FWHM (eV)	At. %	Assignement
	454.2 (460.2)	1.00 (1.50)	34.2	Mo_2TiAlC_2
Ti $2p_{3/2}$ (Ti $2p_{1/2}$)	455.5 (461.5)	2.45 (3.67)	44.2	$Mo_2Ti^{II}AlC_2$
	457.9 (463.9)	2.40 (3.60)	21.6	$Mo_2Ti^{III}AlC_2$
Mo $3d_{5/2}$ (Mo $3d_{3/2}$)	227.7 (230.9)	0.96 (0.96)	100	Mo_2TiAlC_2
	282.4	1.63	22.0	Mo2TiAlC2
C 1s	284.8	1.45	69.0	Adv. C-C
	286.0	1.76	9.0	Adv. C-O
Al $2p_{3/2}$	74.3	2.40	100.0	Mo_2TiAlC_2
O 1s	531.4	2.66	100.0	Adv. O-C

Table S2. Peak positions, full-width at half maximum (FWHM), concentration and assignement for XPS data for sample $Mo_2TiC_2T_x$.

Region	BE (eV)	FWHM (eV)	At. %	Assignement
Ti $2p_{3/2}$ (Ti $2p_{1/2}$)	454.8 (460.8)	1.11 (1.63)	11.1	Mo_2TiAlC_2
	455.4 (461.2)	2.2 (3.3)	35.0	$Mo_2Ti^{II}AlC_2$
	457.6 (463.6)	2.39 (3.58)	21.3	$Mo_2Ti^{III}AlC_2$
	458.6 (464.5)	3.00 (4.50)	32.6	TiO_2
Mo $3d_{5/2}$ (Mo $3d_{3/2}$)	228.0 (231.2)	1.03 (1.03)	100	Mo_2TiAlC_2
C 1s	282.7	1.05	14.6	Mo_2TiAlC_2
	284.8	1.49	70.8	Adv. C-C
	286.0	1.70	9.0	Adv. C-O
	288.2	2.25	5.6	Adv. O-C=O
O 1s	530.2	1.48	45.5	M-O
	531.3	2.36	39.6	Adv. O=C
	533.7	2.58	14.9	Adv. O-C
F 1s	685.1	2.36	100.0	M-F

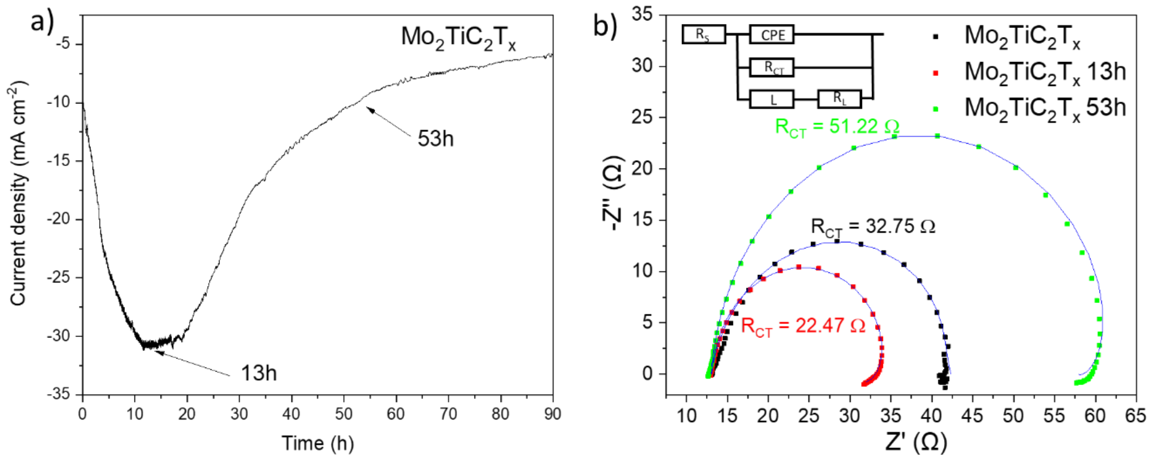

Figure S2. a) Chronoammperomety curve for $Mo_2TiC_2T_x$ sample in 0.5M H_2SO_4 electrolyte. b) Nyquist plots of $Mo_2TiC_2T_x$ sample in 0.5M H_2SO_4 at various times. The blue curve represent a fit of the experimental data.

Table S3. Electrochemical impedance spectroscopy parameters obtained from data fitting.

Sample	R_S (Ω)	R_{CT} (Ω)	R_L (Ω)	Y_{01} ($s^{-n}Ω^{-1}$)	n_1	L_1 (H)
$Mo_2TiC_2T_x$	13.05	32.75	348.90	4.23E-05	0.87	0.72
$Mo_2TiC_2T_x$ 13h	13.07	22.47	82.10	7.12E-05	0.95	0.90
$Mo_2TiC_2T_x$ 53h	13.00	51.22	395.05	4.47E-06	0.94	0.49

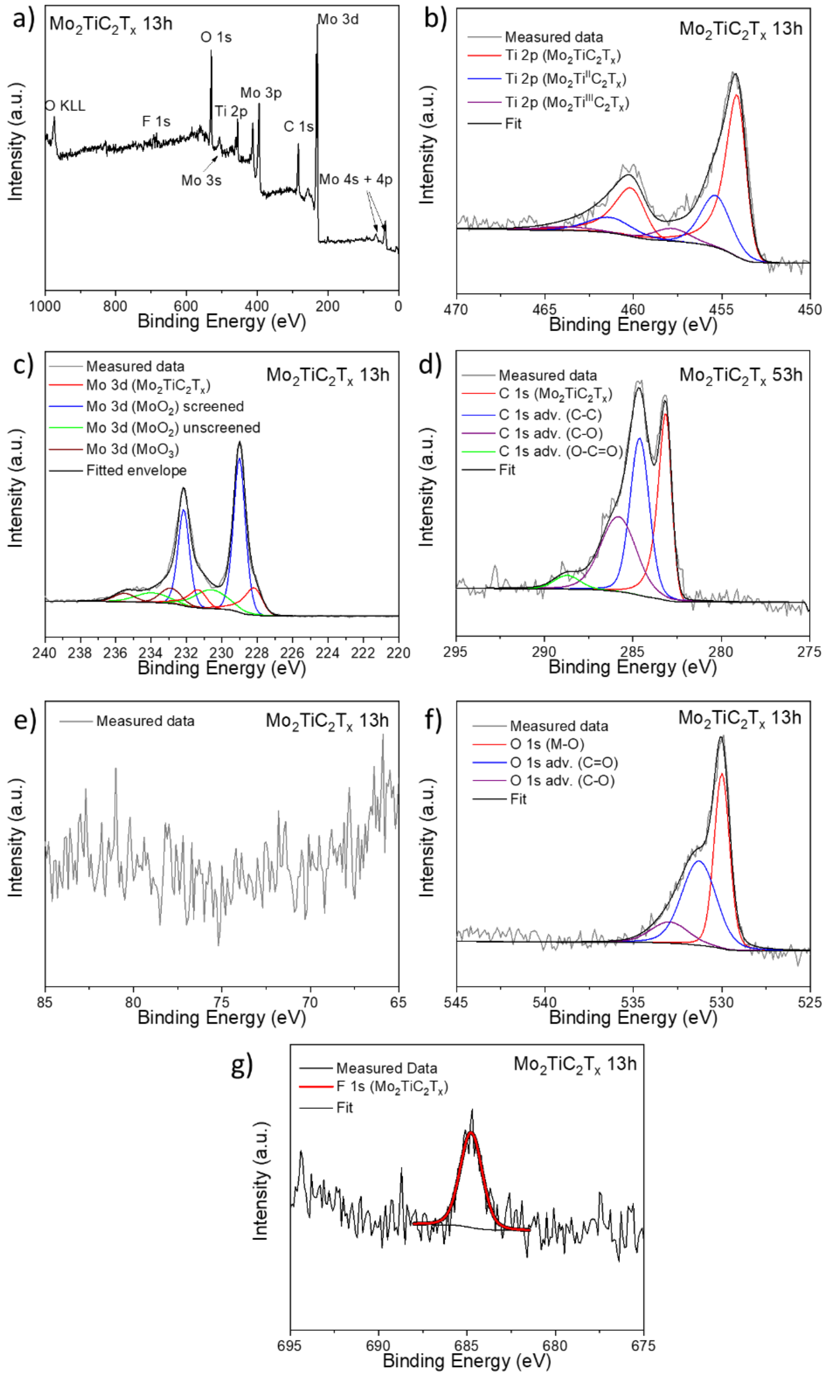

Figure S3. X- ray photoelectron spectra of $Mo_2TiC_2T_x$ 13h sample. a) survey spectrum; b) Ti 2p spectrum; c) Mo 3d spectrum; d) C 1s spectrum; e) Al 2p spectrum, f) O 1s spectrum and g) F 1s spectrum.

Table S4. Peak positions, full-width at half maximum (FWHM), concentration and assignement for XPS data for sample $Mo_2TiC_2T_x$ 13h.

Region	BE (eV)	FWHM (eV)	At. %	Assignement
$Ti\ 2p_{3/2}$ ($Ti\ 2p_{1/2}$)	454.2 (460.2)	1.20 (1.81)	58.2	Mo_2TiAlC_2
	455.4 (461.4)	2.00 (3.0)	32.1	$Mo_2Ti^{II}AlC_2$
	457.8 (463.8)	2.40 (3.60)	9.7	$Mo_2Ti^{III}AlC_2$
$Mo\ 3d_{5/2}$ ($Mo\ 3d_{3/2}$)	228.2 (231.4)	1.08 (1.08)	13.6	Mo_2TiAlC_2
	229.0 (232.2)	0.79 (0.76)	56.5	MoO_2 (screened)
	230.6 (233.9)	2.27 (2.22)	20.0	MoO_2 (unscreened)
	232.9 (235.5)	1.39 (1.39)	9.9	MoO_3
C 1s	283.1	0.82	26.2	Mo_2TiAlC_2
	284.7	1.28	36.9	Adv. C-C
	285.8	2.37	32.6	Adv. C-O
	288.7	1.72	4.3	Adv. O-C=O
O 1s	530.0	1.04	42.5	M-O
	531.3	2.28	45.1	Adv. O=C
	533.0	2.50	12.4	Adv. O-C
F 1s	684.8	1.47	100.0	M-F

Table S5. Peak positions, full-width at half maximum (FWHM), concentration and assignement for XPS data for sample $Mo_2TiC_2T_x$ 53h.

Region	BE (eV)	FWHM (eV)	At. %	Assignement
Ti $2p_{3/2}$ (Ti $2p_{1/2}$)	454.5 (460.5)	1.19 (1.72)	48.1	Mo_2TiAlC_2
	455.3 (461.3)	2.00 (3.00)	39.8	$Mo_2Ti^{II}AlC_2$
	457.4 (463.4)	2.20 (3.30)	12.1	$Mo_2Ti^{III}AlC_2$
Mo $3d_{5/2}$ (Mo $3d_{3/2}$)	228.0 (231.1)	0.70 (0.70)	6.4	Mo_2TiAlC_2
	228.9 (232.1)	0.77 (0.76)	64.1	MoO_2 (screened)
	230.4 (233.8)	2.51 (2.22)	21.1	MoO_2 (unscreened)
	232.9 (235.5)	1.21 (1.21)	8.4	MoO_3
C 1s	283.1	0.93	30.8	Mo_2TiAlC_2
	284.7	1.24	34.3	Adv. C-C
	286.0	2.04	27.6	Adv. C-O
	288.9	1.78	7.3	Adv. O-C=O
O 1s	530.1	0.88	40.5	M-O
	531.3	2.28	41.1	Adv. O=C
	533.3	1.95	18.4	Adv. O-C

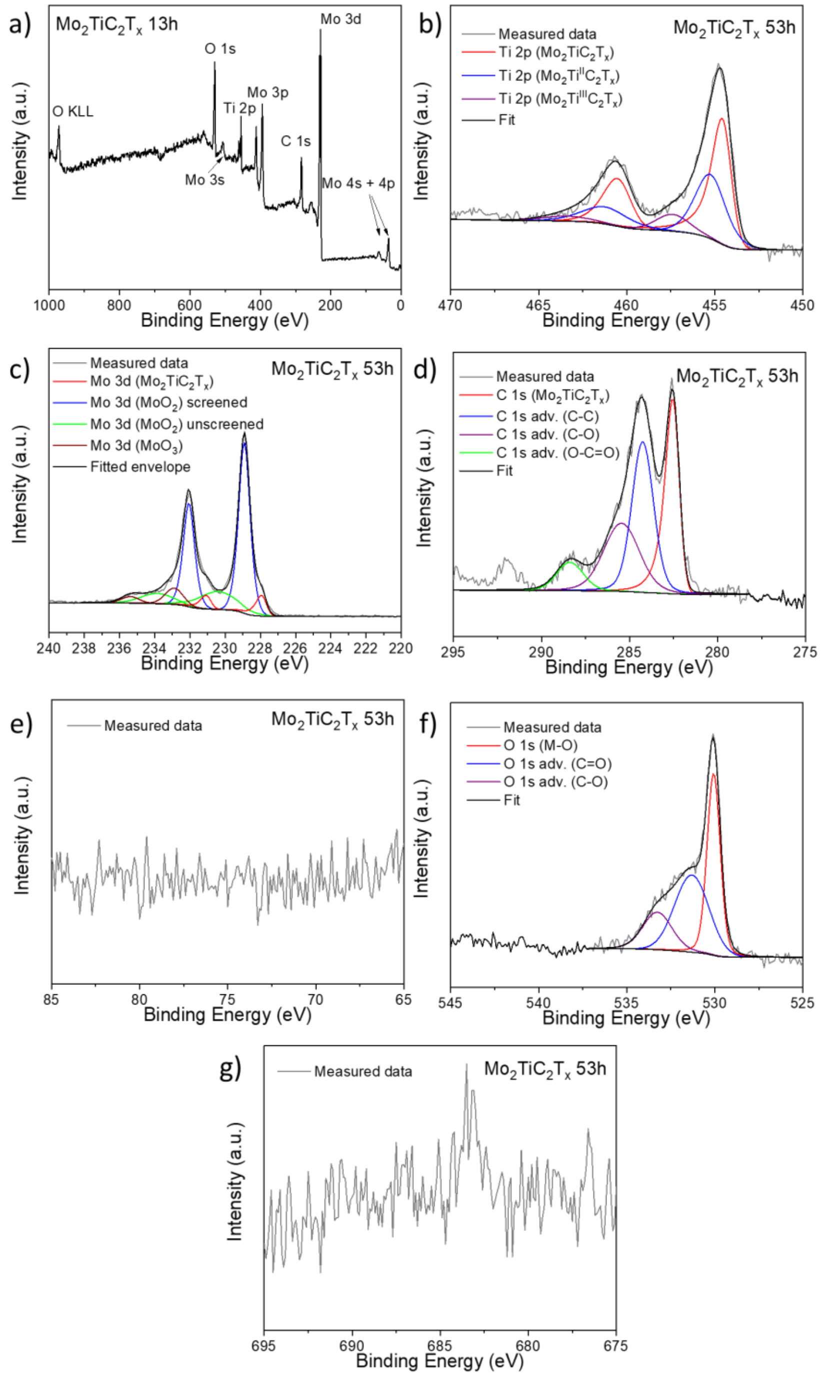

Figure S4. X- ray photoelectron spectra of $Mo_2TiC_2T_x$ 53h sample. a) survey spectrum; b) Ti 2p spectrum; c) Mo 3d spectrum; d) C 1s spectrum; e) Al 2p spectrum, f) O 1s spectrum and g) F 1s spectrum.
